# Senescence: a double-edged sword in beta-cell health and failure?

**DOI:** 10.3389/fendo.2023.1196460

**Published:** 2023-05-09

**Authors:** Sneha S. Varghese, Sangeeta Dhawan

**Affiliations:** Department of Translational Research and Cellular Therapeutics, Arthur Riggs Diabetes and Metabolism Research Institute, City of Hope, Duarte, CA, United States

**Keywords:** beta cells, differentiation, maturation, proliferation, epigenetics, aging, senescence, diabetes

## Abstract

Cellular senescence is a complex process marked by permanent cell-cycle arrest in response to a variety of stressors, and acts as a safeguard against the proliferation of damaged cells. Senescence is not only a key process underlying aging and development of many diseases, but has also been shown to play a vital role in embryogenesis as well as tissue regeneration and repair. In context of the pancreatic beta-cells, that are essential for maintaining glucose homeostasis, replicative senescence is responsible for the age-related decline in regenerative capacity. Stress induced premature senescence is also a key early event underlying beta-cell failure in both type 1 and type 2 diabetes. Targeting senescence has therefore emerged as a promising therapeutic avenue for diabetes. However, the molecular mechanisms that mediate the induction of beta-cell senescence in response to various stressors remain unclear. Nor do we know if senescence plays any role during beta-cell growth and development. In this perspective, we discuss the significance of senescence in beta-cell homeostasis and pathology and highlight emerging directions in this area that warrant our attention.

## 1 Introduction

Cellular senescence is the phenomenon of permanent cell cycle arrest (1) that occurs in response to a variety of stressors, and can serve as a protective mechanism by preventing the proliferation of stressed or damaged cells (2). Senescence plays an important role in embryonic development, tissue regeneration, and repair (3). Senescence is also a fundamental process underlying aging and the pathogenesis of many diseases, including diabetes (4). Accordingly, targeting senescence has emerged as a major therapeutic opportunity in many contexts (5). While the importance of senescence in beta-cell regenerative decline with aging and beta-cell failure in diabetes is firmly established (6–11), we know little about senescence in the context of beta-cell growth and development. As well, the molecular mechanisms that trigger and perpetuate beta-cell senescence remain far from clear. Here, we discuss the role of senescence in beta-cell homeostasis and pathology and highlight key emerging questions.

## 2 The senescence phenotype – a spectrum, not a singularity

**Figure 1:**
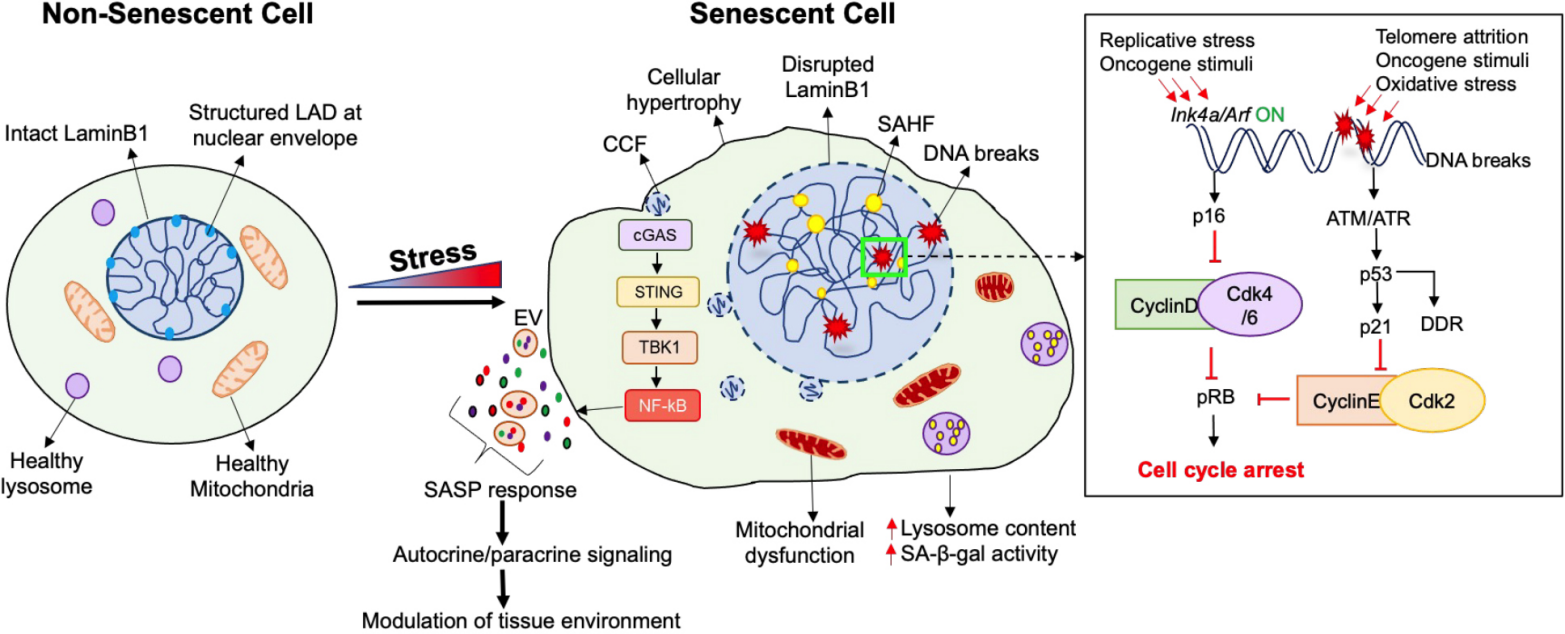
Characteristics of a senescent cell. Senescence, which is a state of irreversible cell cycle arrest, is induced by a variety of stress conditions, resulting in several cellular and morphological changes. Cell cycle arrest in senescence is achieved through the activation of the p16/pRb and/or the p21/p53 pathways, depending upon the type of stress. Apart from cell cycle arrest, the senescent phenotype is heterogenous and may display a combination of morphological features. Overall, a senescent cell (right) exhibits a flattened and hypertrophic morphology compared to a non-senescent cell (left). The structure and function of several organelles is also altered during senescence, such as dysfunctional mitochondria with increased production of Reactive Oxygen Species (ROS), and increased lysosomal number and size along with the induction of a lysosomal senescence associated β-galactosidase (SA-β-gal). In contrast to the intact LaminB1 and structured Lamin Associated-Domains (LADs) in the nucleus of a non-senescent cell, a senescent cell displays an enlarged nucleus with disrupted LaminB1, formation of senescence-associated heterochromatin foci (SAHF), increased DNA breaks, and an elevated DNA damage response (DDR). Senescence can also involve the formation of cytoplasmic chromatin fragments (CCFs) that translocate from the nucleus to the cytosol, and activate the cytosolic DNA sensor cGAS-STING-TBK pathway which further activates the pro-inflammatory senescence associated secretory phenotype (SASP). The SASP response involves direct or extracellular-vesicle (EV) mediated secretion of extracellular matrix modulators, growth factors, cytokines, chemokines, to modulate the tissue micro-environment through autocrine/paracrine signaling.

Senescence is a highly dynamic and heterogenous process, with the phenotype and function of senescent cells unique to the specific inducer and physiologic context (12) (**Figure 1**). Several types of senescence responses have been identified based on the inducing stimulus; these include the classical replicative senescence in response to telomere shortening, oncogene-induced senescence, mitogen-induced senescence, mitochondrial dysfunction associated senescence, and the stress and DNA damage induced premature senescence (5, 12). These stimuli trigger cell-cycle arrest via the activation of two key “tumor-suppressor” modules, namely the p53/p21 and the p16/Rb pathways. Activation of these two pathways propels a variety of the phenotypic changes associated with senescence (2–5, 12–14).

### 2.1 Hallmarks of senescence

Besides replicative arrest and mitogen refractoriness, senescent cells display a variety of phenotypic and molecular hallmarks (12). They often exhibit a flattened and hypertrophic morphology with enlarged nuclei, as well as altered metabolic and mitochondrial activity. Another frequent feature of senescence is lysosomal dysfunction marked by increased senescence-associated β-galactosidase activity (SA-β-gal) (13). Senescent cells also present a hypersecretory phenotype and a characteristic secretory profile consisting of pro-inflammatory cytokines, chemokines, growth factors and proteases, termed the Senescence-Associated Secretory Phenotype (SASP). SASP can attract immune cells such as macrophages, natural killer cells, and T-cells, facilitating immune surveillance and senescent cell elimination (15). Several nuclear changes, such as nuclear LaminB1 depletion and heterochromatic foci, also mark the senescent phenotype (16). Finally, senescent cells exhibit a persistent DNA damage response (DDR) and upregulation of anti-apoptotic programs (2).

### 2.2 Nuclear mechanisms – transcriptional and epigenetic changes

Cell-cycle arrest during senescence primarily involves the activation of either p53/p21 or p16/pRB pathways, or both. p53 and RB serve as transcriptional regulators while p21 and p16 are Cyclin-dependent kinase inhibitors (CDKIs) which mediate cell-cycle arrest (2, 17). The p53/p21 pathway is activated in response to DNA damage caused by telomere attrition, oxidative stress, or oncogenic stimuli (17, 18). A key event in p53 response is the activation of p21, which inhibits CyclinE-Cdk2 complex to mediate cell-cycle arrest. The p53 dependent phosphorylation cascade also stimulates DNA repair and is called the DNA damage response (DDR) (18). Collectively, this response delays cell-cycle until damage is repaired. The RB/p16 pathway, on the other hand, is appropriated not only for oncogene induced senescence, but also during the age-dependent replicative senescence. p16 and p14, both encoded by the *Ink4a/Arf* locus, constitute the cell-cycle inhibitor components of the RB/p16 pathway (17, 19). p16 blocks the formation of the cyclinD-CDK4/6 complexes to prevent RB phosphorylation and promote cell-cycle arrest, while p14 establishes a cross-talk between the p53 and pRB pathways by stabilizing p53 (17).

The transcriptional reprogramming in senescence is assisted by a variety of underlying epigenetic changes. The initial epigenetic changes mediate cell-cycle arrest and establish a pro-survival response to cope with irreparable damage, while the subsequent epigenetic alterations facilitate the pro-inflammatory SASP to modify inter-cellular communication (2). Chromatin changes in senescence involve reduced expression of certain histones and the incorporation of non-canonical histones such as macroH2A. Senescent cells often display a loss of the DNA-nuclear lamina interactions (20). Specifically, the loss of LaminB1 at the Lamina Associated Domains (LADs) leads to heterochromatin redistribution and the formation of senescence-associated heterochromatin foci (SAHF) to facilitate the silencing of proliferation related genes (16).

### 2.3 Cytoplasmic and extracellular responses

DNA damage and genomic instability not only induce changes in gene expression and cell fate, but also trigger an inflammatory response by releasing cytoplasmic chromatin fragments (CCFs) (21). The CCFs are recognized by the cytosolic DNA sensor cyclic GMP-AMP synthase (cGAS), which generates cytosolic GMP-AMP (22). This, in turn, activates the stimulator of interferon genes (STING) and TANK-binding kinase 1 kinase (TBK1), leading to NF-kB activation and the production of inflammatory cytokines and type 1 interferons (23). The CCF-cGAS-STING pathway couples DNA damage sensing to the innate immune response to promote the SASP response (24). SASP modulates the tissue microenvironment and enforces senescence through autocrine and paracrine signaling. The contents of SASP vary based on the cellular context and stress signal (25). This can involve the release of extracellular-vesicles (EVs) which contain nucleic acids such as CCFs along with the pro-inflammatory proteins, to produce long-range effects which can impact distant tissues (26).

### 2.4 Senescence *versus* cell-death – a cell-fate choice

Cells can respond to high levels of stress by triggering senescence or cell-death depending on the specific stressor and cell type, often by repurposing the same pathways (27). Both p53/p21 and Rb/p16 pathways serve pleiotropic roles in this context, and the level and duration of their induction can determine the cell fate choice between apoptosis and senescence. Low levels of p16 induce transient cell-cycle arrest, while high levels trigger senescence (28, 29). Similarly, low levels of p53 promote cell-cycle arrest and senescence, while chronically high p53 levels block pro-senescence signals and promote a pro-apoptotic transcriptional response (30, 31). In contrast to apoptosis, senescence offers cellular viability and paracrine communication to neighboring cells through SASP and thus facilitates adaptation to stress (28).

## 3 Senescence in pancreatic beta-cells – in health and disease

Senescence has traditionally been defined as a permanent loss of replicative capacity and therefore studied predominantly in replication-competent cells. However, senescence is also a multifaceted stress and damage response that initiates as an adaptive process and can turn maladaptive upon prolonged stress (3, 32). Activation of many facets of senescence has been reported in a variety of postmitotic cells including beta-cells (6, 9–11, 33–36), suggesting that senescence in such cells begins as a stress-response (36, 37), temporally separated from permanent replicative arrest. Terminally differentiated cells can enter a senescence-like state from the quiescent G_0_ phase in response to DNA damage or telomere attrition, a phenomenon termed postmitotic cellular senescence (PoMiCS) or ‘amitosenescence’ (34, 38). In some instances, unscheduled re-entry of postmitotic cells into cell-cycle in the context of stress may cause abnormal DNA content and DDR, triggering a senescence like-response labeled as “pseudo-mitosenescence” (38).

### 3.1 Beta-cell senescence – distinct from quiescence

Pancreatic beta-cells transition from a wave of replication dependent expansion in the neonatal growth phase to a functionally mature, postmitotic state in postnatal life marked by a form of cell-cycle exit termed “quiescence”. Quiescence is different from senescence; while quiescence occurs in the G0 phase of cell-cycle, senescence primarily occurs in the G1 and sometimes the G2 phases (12, 39, 40). Furthermore, quiescent cells retain the capacity to re-enter cell-cycle in response to mitogens, unlike senescent cells. The quiescent, postmitotic nature of beta-cells presents a unique scenario in response to stress; it demands robust stress- and pro survival-responses to protect existing cells, while the ability to re-enter cell-cycle offers a way to regenerate beta-cell mass through replication. These two potential responses can create a conflicting choice for beta-cells; to either attempt replication under conditions of stress or activate pro-survival stress-responses. Depending on the severity and duration of the stressor, this may foster successful adaptation, trigger senescence, or induce apoptosis in the most extreme case (3, 32). **Table 1** provides a summary of the unique markers of beta-cell senescence in various contexts.

**Table 1:** Features of beta-cell senescence in different contexts, highlighting the SASP markers unique to beta-cells in each context. M indicated data from mouse models, while Hu indicates data from human samples.

	T1D	T2D/IR	MODY	Aging
**Cell cycle inhibitors**	Cdkn1a, Cdkn2a (M) CDKN1A, CDKN2A (Hu)	Cdkn1a, Cdkn2a (M) *CDKN2A (Hu)*	Cdkn1a (M)	Cdkn1a, Cdkn2a (M)
**Unique SASP markers** **(mRNA/protein)**	IL-6, Igfbp3, Serpine1, Mmp2, Flnb (M) IL-6, SERPINE1 (Hu)	*Gstp1, Gdf15, Hsp90aa1 (M)* *CCL4*, *IL6 (Hu)*	Serpine 1, Cxcl1, Cxcl2, Il6, Tnf (M)	Il6, Tnf and Cxcl1 (M) *Dusp3, Gdf15, Ing1, and Kpnb1 (M)*
**DDR**	γ-H2Ax (M)	53BP1 (Hu)	γ-H2Ax, 53BP1(M)	53BP1 (M)
**Loss of mature beta cell identity**	Yes (M)	Yes (M)	Yes (M)	Yes (M)
**SA β-gal positivity**	Yes (M)	ND	Yes (M)	Yes (M)
**Antiapoptotic** **phenotype**	Yes (M)	Yes (Hu)	Yes (M)	Yes (M)
**Other features**	N/A	*IGF1R* activation	Sex-linked effect of the MODY MAFA S64F mutation, observed in males, Loss of nuclear LaminB1	Sensitive to Cdkn2a targeting
**References**	(9)	(11, 41)	(10)	(11, 41)

IR, insulin resistance; T2D, Type 2 Diabetes; T1D, Type 1 Diabetes.

### 3.2 Replicative senescence in beta-cells – implications for function and regeneration

**Figure 2:**
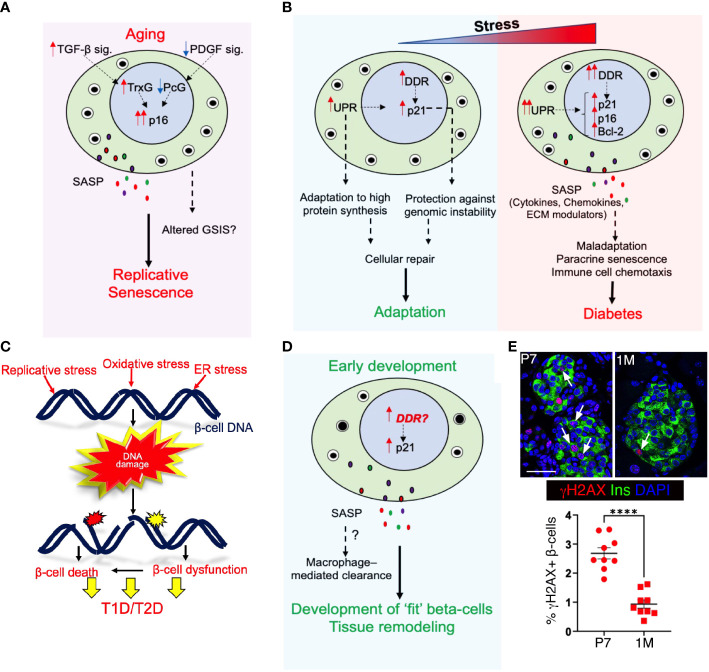
Senescence in pancreatic beta-cell health and disease: **(A)** Aging induces p16 accumulation and leads to replicative senescence, which limits beta-cell self-renewal. Age associated transcriptional changes may also alter basal insulin secretion without any accompanying changes in glucose stimulated insulin secretion (GSIS). With aging, reduced Platelet Derived Growth Factor signaling (PDGF sig.) and increased Transforming Growth Factor-beta signaling (TGFb sig.) leads to alleviation of Polycomb (PcG)- mediated repression of p16 locus and drives Trithorax (TrxG)-mediated activation of p16 locus. **(B)** The duration or intensity of exposure to cellular-stress stimuli dictates the beta-cell response; either facilitating adaptation or causing maladaptation. Exposure to transient stress initiates DDR and unfolded protein response (UPR) and facilitates cellular repair and adaptation. However, prolonged stress exposure aggravates both DDR and UPR and induces SASP, leading to premature senescence. This can cause beta-cell dysfunction and maladaptation, and pre-dispose to diabetes. **(C)** Both intrinsic and extrinsic stress stimuli can induce DNA damage in beta-cells through a myriad of triggers such as replicative stress, ROS, and ER-stress, and result in beta-cell dysfunction and/or death. **(D)** We propose that unresolved DNA damage in the developing postnatal pancreas may trigger a p21 response and SASP to mediate the macrophage-mediated clearance of damaged beta-cells during this phase, allowing only health and fit beta-cells to mature. Our data shown in **(E)** imply that indeed the early postnatal beta-cell expansion phase is vulnerable to DNA damage accumulation. Top panel shows representative images of wild-type mouse pancreatic sections at postnatal day 7 (p7) and I month (1m), immuno-stained for the DNA damage marker γH2AX (red) along with Insulin (green) and DAPI (blue), while the lower panel show a quantification of % γH2AX+ beta-cells in the two stages, pointing to high DNA damage vulnerability in early postnatal beta-cells. Error-bars show SEM. ****P<0.001, determined by using a two-tailed Student’s t-test . Scale bar: 50 mm.

Adult beta-cells can expand by replication to adapt to increased insulin demand due to injury or insulin resistance. However, their expansion capacity declines with age due to replicative senescence accompanied by the epigenetically controlled, gradual accumulation of p16 (6–8, 42, 43). In young beta-cells, Polycomb proteins Ezh2 and Bmi1 repress the *p16* locus. The levels of Ezh2 gradually decline with aging, resulting in reduced binding of both polycomb proteins and concomitant reduction of repressive chromatin modifications (7, 8). This is accompanied by an age-related increase in binding of the Trithorax complex containing Mll1 and JmjD3 which mark the chromatin with activating histone modifications, leading to p16 accumulation (44). The epigenetic control of p16 is driven by age-related changes in growth-factor signaling pathways (45). Platelet-derived Growth Factor (PDGF) signaling, which is essential for Ezh2 expression, declines with aging (46). On the other hand, age-related increase in Transforming Growth Factor-beta (TGFb) signaling promote Trithorax complex recruitment to the *Ink4a/Arf* locus, thus contributing to the onset of replicative senescence (47) (**Figure 2A**).

The induction of p16 in adult beta-cells is first observed around 2.5 months of age in mice (8), and appears to occur as a part of their normal functional maturation (48). In agreement, ectopic expression of p16 in beta-cells of juvenile mice not only induces replicative senescence but also enhances GSIS (48). This agrees with data in murine islets showing the epigenetic activation of transcriptional programs underlying insulin secretion with age, while the genes related to beta-cell replication are epigenetically repressed (49). In contrast, plenty of evidence suggests that basal insulin secretion increases with aging, accompanied by either unaltered or increased stimulated insulin secretion (50). It is possible that p16 represents only a part of the replicative senescence program, and other mechanisms are maybe involved in the age-related changes in glucose responsiveness and insulin secretion. The presence of p16 in beta-cells of young mice points to that (8, 48), and suggests that p16 expression alone may not indicate replicative senescence. Alternatively, the levels and duration of p16 expression may account for these age-related differences in beta-cell phenotype. In agreement, many key hallmarks of senescence, such as SA-β-gal only appear in islets of really old mice (51). Furthermore, a transcriptomic comparison of SA-β-gal+ and SA-β-gal- beta-cells showed impaired glucose-sensing machinery in the senescent cells (11). Replicative senescence prevents the replication of cells that have accumulated damage with age, while the concomitant hypersecretory phenotype could allow beta-cells to compensate for an acute and modest increased insulin demand. However, this may not suffice for chronically high insulin requirement and can cause impaired glucose homeostasis, as has been observed in old mice in many contexts that warrant beta-cell expansion (42, 43, 52).

### 3.3 Stress-induced senescence in beta-cells – relevance for diabetes

Beta-cell fragility is a shared underlying feature of both type 1- and type 2- diabetes (T1D and T2D), with the activation of unfolded protein response (UPR) and DDR preceding beta-cell failure (53–57). In addition, the immune-mediated islet inflammation is a well-established stress trigger in T1D and is now also recognized to contribute to beta-cell failure in T2D (58, 59). One of the key sequalae of beta-cell stress in both T1D and T2D is the activation of premature senescence involving both p16 and p21, DDR, and SASP, along with the induction of Bcl-2 as a pro-survival mechanism (9, 11). The beta-cell SASP secretome in diabetes not only harbors inflammatory cytokines and chemokines, but also includes extracellular matrix modulators such as Mmp2, Serpine1, Igfbp3, and FilaminB (9). The SASP response displays non-cell-autonomous activities, such as paracrine senescence and promoting the chemotaxis of immune cells (60, 61).

Besides the immune and metabolic factors as triggers for beta-cell stress (57, 62, 63), the contribution of beta-cell intrinsic changes is becoming increasingly evident (64). For instance, genetic vulnerability due to mutations in DNA repair genes can trigger beta-cell failure independent of immune defects (53). Beta-cells can accumulate oxidative-stress induced DNA damage and somatic mutations, which could predispose to senescence (65). More recently, a missense mutation in the beta-cell transcription factor MafA associated with a form of maturity onset diabetes of the young (MODY) was shown to induce premature senescence and SASP (10). These data reiterate the involvement of beta-cell intrinsic triggers for SASP in diabetes. Given the role of MafA in mature beta-cell identity, this also suggests a link between impaired beta-cell identity and senescence. In agreement, beta-cells harboring SASP signatures in T1D display reduced levels of the maturity marker Ucn3 (9). Similarly, senescent beta-cells in the context of insulin resistance display a loss of the mature beta-cell identity (11). Thus, beta-cell intrinsic factors such as loss of mature identity and impaired stress-response may be critical in initiating senescence.

Whether a beta-cell adapts or fails in response to stress depends on the severity and duration of the stressor, as well as its intrinsic capacity to handle stress (**Figure 2B**). Different beta-cell subtypes display differential stress-responsiveness and predisposition to senescence (11, 51, 66–68). Stress-induced changes in beta-cell identity and heterogeneity (69–73) can therefore impair the collective stress responsiveness of the beta-cell pool and induce senescence. Indeed, increased UPR and DDR precede senescence and have an additive effect towards inducing senescence (74–76). UPR initiates as an adaptive response and allows cells to cope with a high demand for protein synthesis and processing, while DDR protects against genomic instability due to replication stress and oxidative stress (77, 78). The link between UPR, DDR, and senescence is especially relevant for beta-cells that produce and process a large amount of protein load, rely on replication for adaptive expansion, and are highly susceptible to oxidative damage (79–81) (**Figure 2C**). While the initial induction of these stress-sensing adaptive mechanisms in response to stress facilitates cellular repair and adaptation, persistent activation of these mechanisms leads to tissue maladaptation, senescence, and even death (53–57). Recent data show that p21 is a temporal stress-sensor that shapes the cellular response by placing cells under immunosurveillance, which either disengages or eliminates damaged cells depending on the duration of p21 persistence (82). This suggests that the duration of such responses can dictate whether cells undergo repair, become senescent, or undergo cell death, effectively determining the choice between adaptation vs maladaptation.

### 3.4 Senescence in beta-cell development – making “fit” beta-cells

Senescence is also known to occur during embryonic development and is essential for growth and patterning. Such developmentally programmed senescence relies on p21 activation and SASP, which induces macrophage mediated clearance of senescent cells and contributes to tissue remodeling (83–86). Macrophage infiltration is observed in both mice and humans during pancreas development (87–90), and regulates pancreatic progenitor migration, cell cycle progression, and beta-cell expansion (91, 92). Macrophages likely also assist the clearance of damaged cells that may accumulate due to the extensive cell turnover during pancreas development (93–96). While prior work on developmental senescence showed that p21 induction did not involve DNA damage and p53 activation (85, 86), the scenario in the maturing pancreas might be different (**Figure 2D**). High rates of beta-cell replication during postnatal growth appear to increase vulnerability to DNA damage (**Figure 2E**). Beta-cells with extensive DNA damage could then initiate a SASP response towards their clearance by macrophages, allowing only “fit” beta-cells to mature and establish a healthy beta-cell pool. Alternatively, as a recent lineage tracing study suggests, some senescent cells may resolve senescence to re-enter cell-cycle during development, and progress to maturation (97, 98). Impaired clearance or aberrant cell-cycle entry of damaged beta-cells during development due to any genetic or epigenetic causes could therefore predispose to impaired beta-cell mass and future disease vulnerability (99). Indeed, peak incidence of the development of islet autoimmunity in T1D occurs during the growth phase (100–102). Recent evidence shows that maternal diabetes induces premature senescence in the neuroepithelium, leading to neural tube defects (103). Similar events may occur during beta-cell differentiation and result in impaired beta-cell mass. Investigating senescence during islet morphogenesis is therefore important to identify the triggering events underlying beta-cell pathologies.

### 3.5 Targeting senescence – beta cell therapies

Administration of senolytic agents has been shown to improve glucose homeostasis in mouse models T2D and T1D (9, 11, 104, 105). Inhibitors of the pro-survival Bcl2, such as ABT-199 or ABT-737, can induce apoptosis of senescent beta-cells in the prediabetic Non-obese diabetic (NOD) mice, and reduce the expression of SASP markers *in vivo* (9). Similarly, Bcl2 targeting using ABT-263 has also been shown to partially restore beta-cell mature phenotype and reverse metabolic defects in both acute and chronic insulin resistance (11). The bromodomain extra-terminal (BET) domain proteins, key inducers of SASP in T1D, are another emerging target whose inhibition using iBET-762 prevents SASP and autoimmune diabetes in the NOD mice (104). Transcriptomic profiling of senescent beta-cells in the context of insulin resistance shows potential of targeting the HIF1a pathway using desatinib and quercetin (D+Q) (11), a senolytic strategy that is in phase-I trials for other diseases (106). While senolytics are highly promising, they may have off-target effects (107). Moreover, the heterogeneous frequency of senescent beta-cells in pre-diabetes precludes the identification of patients who would benefit the most from senolytics (9). Development of beta-cell targeting approaches and serum biomarkers for SASP would facilitate the optimization of senolytics for clinical translation.

## 4 Summary and emerging directions

Beta-cells undergo profound phenotypic changes in diabetes, recapitulating several features of the functionally immature fetal and neonatal beta-cells. These changes likely initiate as an adaptation to stress, to protect cells from damage and promote repair and regeneration. With chronic stress, attempted regeneration and increased functional workload can aggravate genotoxic and proteotoxic stress, and result in senescence and maladaptation. Moreover, any defects in stress-response during beta-cell growth in early life can set the stage for future beta-cell failure. Therefore, it is essential to identify mechanisms that define the mature beta-cell phenotype, protect beta-cell genomic stability, and are altered in response to genotoxic and metabolic stress as beta-cells adapt or fail. These may include critical growth signals and epigenetic mechanisms that program the mature beta-cell transcriptional landscape. Stress-responsive modulators of chromatin 3D architecture such as the cohesin complex and the CCCTC binding factor (CTCF) that are essential for genomic stability and transcriptional control may be suitable candidates for such studies (108–111). Another critical question is the link between senescence and the islet-immune interaction during development and diabetes. It would also be pertinent to compare the beta-cell SASP in aging, T1D, and T2D to determine its context specificity. Understanding the mechanisms that safeguard beta-cell genomic stability and stress-response and their impact on replicative and stress-induced senescence programs will be key to identifying the molecular triggers of beta-cell failure in diabetes.

## Data availability statement

The raw data supporting the conclusions of this article will be made available by the authors, without undue reservation.

## Ethics statement

The animal study was reviewed and approved by Institutional Animal Care and Use Committee (IACUC) at City of Hope.

## Author contributions

Both SV and SD contributed to the conception of the article layout, critical review of the literature, preparation and editing of the manuscript contents, and approving the final version for publication.

## References

[1] Hayflick L Moorhead PS. The serial cultivation of human diploid cell strains. Exp Cell Res (1961) 25:585–621. doi: 10.1016/0014-4827(61)90192-6 13905658

[2] Herranz N Gil J. Mechanisms and functions of cellular senescence. J Clin Invest (2018) 128(4):1238–46. doi: 10.1172/JCI95148 PMC5873888 29608137

[3] Paramos-de-Carvalho D Jacinto A Saude L. The right time for senescence. Elife (2021) 10. doi: 10.7554/eLife.72449 PMC8580479 34756162

[4] Childs BG Durik M Baker DJ van Deursen JM. Cellular senescence in aging and age-related disease: from mechanisms to therapy. Nat Med (2015) 21(12):1424–35. doi: 10.1038/nm.4000 PMC4748967 26646499

[5] Di Micco R Krizhanovsky V Baker D d’Adda di Fagagna F. Cellular senescence in ageing: from mechanisms to therapeutic opportunities. Nat Rev Mol Cell Biol (2021) 22(2):75–95. doi: 10.1038/s41580-020-00314-w 33328614 PMC8344376

[6] Krishnamurthy J Ramsey MR Ligon KL Torrice C Koh A Bonner-Weir S. p16INK4a induces an age-dependent decline in islet regenerative potential. Nature (2006) 443(7110):453–7. doi: 10.1038/nature05092 16957737

[7] Chen H Gu X Su IH Bottino R Contreras JL Tarakhovsky A. Polycomb protein Ezh2 regulates pancreatic beta-cell Ink4a/Arf expression and regeneration in diabetes mellitus. Genes Dev (2009) 23(8):975–85. doi: 10.1101/gad.1742509 PMC2675862 19390090

[8] Dhawan S Tschen SI Bhushan A. Bmi-1 regulates the Ink4a/Arf locus to control pancreatic beta-cell proliferation. Genes Dev (2009) 23(8):906–11. doi: 10.1101/gad.1742609 PMC2675870 19390085

[9] Thompson PJ Shah A Ntranos V Van Gool F Atkinson M Bhushan A. Targeted elimination of senescent beta cells prevents type 1 diabetes. Cell Metab (2019) 29(5):1045–60 e10. doi: 10.1016/j.cmet.2019.01.021 30799288

[10] Walker EM Cha J Tong X Guo M Liu JH Yu S. Sex-biased islet beta cell dysfunction is caused by the MODY MAFA S64F variant by inducing premature aging and senescence in males. Cell Rep (2021) 37(2):109813. doi: 10.1016/j.celrep.2021.109813 34644565 PMC8845126

[11] Aguayo-Mazzucato C Andle J Lee TB Jr. Midha A Talemal L Chipashvili V. Acceleration of beta cell aging determines diabetes and senolysis improves disease outcomes. Cell Metab (2019) 30(1):129–42 e4. doi: 10.1016/j.cmet.2019.05.006 31155496 PMC6610720

[12] Kumari R Jat P. Mechanisms of cellular senescence: cell cycle arrest and senescence associated secretory phenotype. Front Cell Dev Biol (2021) 9:645593. doi: 10.3389/fcell.2021.645593 33855023 PMC8039141

[13] Hernandez-Segura A Nehme J Demaria M. Hallmarks of cellular senescence. Trends Cell Biol (2018) 28(6):436–53. doi: 10.1016/j.tcb.2018.02.001 29477613

[14] Salama R Sadaie M Hoare M Narita M. Cellular senescence and its effector programs. Genes Dev (2014) 28(2):99–114. doi: 10.1101/gad.235184.113 24449267 PMC3909793

[15] Faget DV Ren Q Stewart SA. Unmasking senescence: context-dependent effects of SASP in cancer. Nat Rev Cancer (2019) 19(8):439–53. doi: 10.1038/s41568-019-0156-2 31235879

[16] Rocha A Dalgarno A Neretti N. The functional impact of nuclear reorganization in cellular senescence. Brief Funct Genomics (2022) 21(1):24–34. doi: 10.1093/bfgp/elab012 33755107 PMC8789270

[17] Kumari R Hummerich H Shen X Fischer M Litovchick L Mittnacht S. Simultaneous expression of MMB-FOXM1 complex components enables efficient bypass of senescence. Sci Rep (2021) 11(1):21506. doi: 10.1038/s41598-021-01012-z 34728711 PMC8563780

[18] Mijit M Caracciolo V Melillo A Amicarelli F Giordano A. Role of p53 in the regulation of cellular senescence. Biomolecules (2020) 10(3). doi: 10.3390/biom10030420 PMC7175209 32182711

[19] Rattanavirotkul N Kirschner K Chandra T. Induction and transmission of oncogene-induced senescence. Cell Mol Life Sci (2021) 78(3):843–52. doi: 10.1007/s00018-020-03638-0 PMC7897614 32936311

[20] Paluvai H Di Giorgio E Brancolini C. The histone code of senescence. Cells (2020) 9(2). doi: 10.3390/cells9020466 PMC7072776 32085582

[21] Dou Z Ghosh K Vizioli MG Zhu J Sen P Wangensteen KJ. Cytoplasmic chromatin triggers inflammation in senescence and cancer. Nature (2017) 550(7676):402–6. doi: 10.1038/nature24050 PMC5850938 28976970

[22] Yang H Wang H Ren J Chen Q Chen ZJ. cGAS is essential for cellular senescence. Proc Natl Acad Sci USA (2017) 114(23):E4612–E20. doi: 10.1073/pnas.1705499114 PMC5468617 28533362

[23] Hopfner KP Hornung V. Molecular mechanisms and cellular functions of cGAS-STING signalling. Nat Rev Mol Cell Biol (2020) 21(9):501–21. doi: 10.1038/s41580-020-0244-x 32424334

[24] Li T Chen ZJ. The cGAS-cGAMP-STING pathway connects DNA damage to inflammation, senescence, and cancer. J Exp Med (2018) 215(5):1287–99. doi: 10.1084/jem.20180139 PMC5940270 29622565

[25] Hoare M Ito Y Kang TW Weekes MP Matheson NJ Patten DA. NOTCH1 mediates a switch between two distinct secretomes during senescence. Nat Cell Biol (2016) 18(9):979–92. doi: 10.1038/ncb3397 PMC5008465 27525720

[26] Takasugi M. Emerging roles of extracellular vesicles in cellular senescence and aging. Aging Cell (2018) 17(2). doi: 10.1111/acel.12734 PMC5847882 29392820

[27] Vicencio JM Galluzzi L Tajeddine N Ortiz C Criollo A Tasdemir E. Senescence, apoptosis or autophagy? when a damaged cell must decide its path–a mini-review. Gerontology (2008) 54(2):92–9. doi: 10.1159/000129697 18451641

[28] Childs BG Baker DJ Kirkland JL Campisi J van Deursen JM. Senescence and apoptosis: dueling or complementary cell fates? EMBO Rep (2014) 15(11):1139–53. doi: 10.15252/embr.201439245 PMC4253488 25312810

[29] Liu JY Souroullas GP Diekman BO Krishnamurthy J Hall BM Sorrentino JA. Cells exhibiting strong p16(INK4a) promoter activation *in vivo* display features of senescence. Proc Natl Acad Sci USA (2019) 116(7):2603–11. doi: 10.1073/pnas.1818313116 PMC6377452 30683717

[30] Chen J. The cell-cycle arrest and apoptotic functions of p53 in tumor initiation and progression. Cold Spring Harb Perspect Med (2016) 6(3):a026104. doi: 10.1101/cshperspect.a026104 26931810 PMC4772082

[31] Rizzotto D Englmaier L Villunger A. At A crossroads to cancer: how p53-induced cell fate decisions secure genome integrity. Int J Mol Sci (2021) 22(19). doi: 10.3390/ijms221910883 PMC8509445 34639222

[32] Kirschner K Rattanavirotkul N Quince MF Chandra T. Functional heterogeneity in senescence. Biochem Soc Trans (2020) 48(3):765–73. doi: 10.1042/BST20190109 PMC7329341 32369550

[33] von Zglinicki T Wan T Miwa S. Senescence in post-mitotic cells: a driver of aging? Antioxid Redox Signal (2021) 34(4):308–23. doi: 10.1042/BST20190109 PMC7821432 32164429

[34] Sapieha P Mallette FA. Cellular senescence in postmitotic cells: beyond growth arrest. Trends Cell Biol (2018) 28(8):595–607. doi: 10.1016/j.tcb.2018.03.003 29704982

[35] Herdy JR Traxler L Agarwal RK Karbacher L Schlachetzki JCM Boehnke L. Increased post-mitotic senescence in aged human neurons is a pathological feature of alzheimer’s disease. Cell Stem Cell (2022) 29(12):1637–52.e6. doi: 10.1016/j.stem.2022.11.010 36459967 PMC10093780

[36] Anderson R Lagnado A Maggiorani D Walaszczyk A Dookun E Chapman J. Length-independent telomere damage drives post-mitotic cardiomyocyte senescence. EMBO J (2019) 38(5). doi: 10.15252/embj.2018100492 PMC6396144 30737259

[37] Jurk D Wang C Miwa S Maddick M Korolchuk V Tsolou A. Postmitotic neurons develop a p21-dependent senescence-like phenotype driven by a DNA damage response. Aging Cell (2012) 11(6):996–1004. doi: 10.1111/j.1474-9726.2012.00870.x 22882466 PMC3533793

[38] Wengerodt D Schmeer C Witte OW Kretz A. Amitosenescence and pseudomitosenescence: putative new players in the aging process. Cells (2019) 8(12). doi: 10.3390/cells8121546 PMC6952980 31795499

[39] Terzi MY Izmirli M Gogebakan B. The cell fate: senescence or quiescence. Mol Biol Rep (2016) 43(11):1213–20. doi: 10.1007/s11033-016-4065-0 27558094

[40] Fujimaki K Yao G. Cell dormancy plasticity: quiescence deepens into senescence through a dimmer switch. Physiol Genomics (2020) 52(11):558–62. doi: 10.1152/physiolgenomics.00068.2020 32986540

[41] Midha A Pan H Abarca C Andle J Carapeto P Bonner-Weir S. Unique human and mouse beta-cell senescence-associated secretory phenotype (sasp) reveal conserved signaling pathways and heterogeneous factors. Diabetes (2021) 70(5):1098–116. doi: 10.2337/db20-0553 PMC8173799 33674410

[42] Tschen SI Dhawan S Gurlo T Bhushan A. Age-dependent decline in beta-cell proliferation restricts the capacity of beta-cell regeneration in mice. Diabetes (2009) 58(6):1312–20. doi: 10.2337/db08-1651 PMC2682690 19228811

[43] Rankin MM Kushner JA. Adaptive beta-cell proliferation is severely restricted with advanced age. Diabetes (2009) 58(6):1365–72. doi: 10.2337/db08-1198 PMC2682671 19265026

[44] Zhou JX Dhawan S Fu H Snyder E Bottino R Kundu S. Combined modulation of polycomb and trithorax genes rejuvenates beta cell replication. J Clin Invest (2013) 123(11):4849–58. doi: 10.1172/JCI69468 PMC3809789 24216481

[45] Varghese SS Dhawan S. Polycomb repressive complexes: shaping pancreatic beta-cell destiny in development and metabolic disease. Front Cell Dev Biol (2022) 10:868592. doi: 10.3389/fcell.2022.868592 35602600 PMC9116887

[46] Chen H Gu X Liu Y Wang J Wirt SE Bottino R. PDGF signalling controls age-dependent proliferation in pancreatic beta-cells. Nature (2011) 478(7369):349–55. doi: 10.1038/nature10502 PMC3503246 21993628

[47] Dhawan S Dirice E Kulkarni RN Bhushan A. Inhibition of TGF-beta signaling promotes human pancreatic beta cell replication. Diabetes (2016) 65(5):1208–18. doi: 10.2337/db15-1331 PMC4839200 26936960

[48] Helman A Klochendler A Azazmeh N Gabai Y Horwitz E Anzi S. p16(Ink4a)-induced senescence of pancreatic beta cells enhances insulin secretion. Nat Med (2016) 22(4):412–20. doi: 10.1038/nm.4054 PMC5546206 26950362

[49] Avrahami D Li C Zhang J Schug J Avrahami R Rao S. Aging-dependent demethylation of regulatory elements correlates with chromatin state and improved beta cell function. Cell Metab (2015) 22(4):619–32 doi: 10.1016/j.cmet.2015.07.025 PMC4598285 26321660

[50] Aguayo-Mazzucato C. Functional changes in beta cells during ageing and senescence. Diabetologia (2020) 63(10):2022–9. doi: 10.1007/s00125-020-05185-6 PMC7990033 32894312

[51] Aguayo-Mazzucato C van Haaren M Mruk M Lee TB Jr. Crawford C Hollister-Lock J. Beta cell aging markers have heterogeneous distribution and are induced by insulin resistance. Cell Metab (2017) 25(4):898–910 e5. doi: 10.1016/j.cmet.2017.03.015 28380379 PMC5471618

[52] Teta M Long SY Wartschow LM Rankin MM Kushner JA. Very slow turnover of beta-cells in aged adult mice. Diabetes (2005) 54(9):2557–67. doi: 10.2337/diabetes.54.9.2557 16123343

[53] Dooley J Tian L Schonefeldt S Delghingaro-Augusto V Garcia-Perez JE Pasciuto E. Genetic predisposition for beta cell fragility underlies type 1 and type 2 diabetes. Nat Genet (2016) 48(5):519–27. doi: 10.1038/ng.3531 PMC5584070 26998692

[54] Horwitz E Krogvold L Zhitomirsky S Swisa A Fischman M Lax T. Beta-cell DNA damage response promotes islet inflammation in type 1 diabetes. Diabetes (2018) 67(11):2305–18. doi: 10.2337/db17-1006 PMC6198335 30150306

[55] Tersey SA Nishiki Y Templin AT Cabrera SM Stull ND Colvin SC. Islet beta-cell endoplasmic reticulum stress precedes the onset of type 1 diabetes in the nonobese diabetic mouse model. Diabetes (2012) 61(4):818–27. doi: 10.2337/db11-1293 PMC3314371 22442300

[56] Tornovsky-Babeay S Dadon D Ziv O Tzipilevich E Kadosh T Schyr-Ben Haroush R. Type 2 diabetes and congenital hyperinsulinism cause DNA double-strand breaks and p53 activity in beta cells. Cell Metab (2014) 19(1):109–21. doi: 10.1016/j.cmet.2013.11.007 24332968

[57] Kulkarni A Muralidharan C May SC Tersey SA Mirmira RG. Inside the beta cell: molecular stress response pathways in diabetes pathogenesis. Endocrinology (2022) 164(1). doi: 10.1210/endocr/bqac184 PMC9667558 36317483

[58] Brooks-Worrell B Hampe CS Hattery EG Palomino B Zangeneh SZ Utzschneider K. Islet autoimmunity is highly prevalent and associated with diminished beta-cell function in patients with type 2 diabetes in the grade study. Diabetes (2022) 71(6):1261–71. doi: 10.2337/db21-0590 PMC9375448 35061024

[59] Yang B Maddison LA Zaborska KE Dai C Yin L Tang Z. RIPK3-mediated inflammation is a conserved beta cell response to ER stress. Sci Adv (2020) 6(51). doi: 10.1126/sciadv.abd7272 PMC11206196 33355143

[60] Acosta JC Banito A Wuestefeld T Georgilis A Janich P Morton JP. A complex secretory program orchestrated by the inflammasome controls paracrine senescence. Nat Cell Biol (2013) 15(8):978–90. doi: 10.1038/ncb2784 PMC3732483 23770676

[61] Kale A Sharma A Stolzing A Desprez PY Campisi J. Role of immune cells in the removal of deleterious senescent cells. Immun Ageing (2020) 17:16. doi: 10.1186/s12979-020-00187-9 32518575 PMC7271494

[62] Atkinson MA Bluestone JA Eisenbarth GS Hebrok M Herold KC Accili D. How does type 1 diabetes develop?: the notion of homicide or beta-cell suicide revisited. Diabetes (2011) 60(5):1370–9. doi: 10.2337/db10-1797 PMC3292309 21525508

[63] Wilcox NS Rui J Hebrok M Herold KC. Life and death of beta cells in type 1 diabetes: a comprehensive review. J Autoimmun (2016) 71:51–8. doi: 10.1016/j.jaut.2016.02.001 PMC4903951 27017348

[64] Roep BO Thomaidou S van Tienhoven R Zaldumbide A. Type 1 diabetes mellitus as a disease of the beta-cell (do not blame the immune system)? Nat Rev Endocrinol (2020) 17:150–61. doi: 10.1038/s41574-020-00443-4 PMC7722981 33293704

[65] Enge M Arda HE Mignardi M Beausang J Bottino R Kim SK. Single-cell analysis of human pancreas reveals transcriptional signatures of aging and somatic mutation patterns. Cell (2017) 171(2):321–30 e14. doi: 10.1016/j.cell.2017.09.004 28965763 PMC6047899

[66] Xin Y Dominguez Gutierrez G Okamoto H Kim J Lee AH Adler C. Pseudotime ordering of single human beta-cells reveals states of insulin production and unfolded protein response. Diabetes (2018) 67(9):1783–94. doi: 10.2337/db18-0365 29950394

[67] Baron M Veres A Wolock SL Faust AL Gaujoux R Vetere A. A single-cell transcriptomic map of the human and mouse pancreas reveals inter- and intra-cell population structure. Cell Syst (2016) 3(4):346–60.e4. doi: 10.1016/j.cels.2016.08.011 27667365 PMC5228327

[68] Muraro MJ Dharmadhikari G Grun D Groen N Dielen T Jansen E. A single-cell transcriptome atlas of the human pancreas. Cell Syst (2016) 3(4):385–94 e3. doi: 10.1016/j.cels.2016.09.002 27693023 PMC5092539

[69] Parveen N Wang JK Bhattacharya S Cuala J Singh Rajkumar M Butler AE. DNA Methylation dependent restriction of tyrosine hydroxylase contributes to pancreatic beta-cell heterogeneity. Diabetes (2023) 72(5):575–89. doi: 10.2337/db22-0506 PMC10130487 36607262

[70] Talchai C Xuan S Lin HV Sussel L Accili D. Pancreatic beta cell dedifferentiation as a mechanism of diabetic beta cell failure. Cell (2012) 150(6):1223–34. doi: 10.1016/j.cell.2012.07.029 PMC3445031 22980982

[71] Dahan T Ziv O Horwitz E Zemmour H Lavi J Swisa A. Pancreatic beta-cells express the fetal islet hormone gastrin in rodent and human diabetes. Diabetes (2017) 66(2):426–36. doi: 10.2337/db16-0641 PMC5248995 27864307

[72] Swisa A Glaser B Dor Y. Metabolic stress and compromised identity of pancreatic beta cells. Front Genet (2017) 8:21. doi: 10.3389/fgene.2017.00021 28270834 PMC5318414

[73] Rodnoi P Rajkumar M Moin ASM Georgia SK Butler AE Dhawan S. Neuropeptide y expression marks partially differentiated beta cells in mice and humans. JCI Insight (2017) 2(12). doi: 10.1172/jci.insight.94005 PMC5470892 28614797

[74] Acosta-Alvear D Zhou Y Blais A Tsikitis M Lents NH Arias C. XBP1 controls diverse cell type- and condition-specific transcriptional regulatory networks. Mol Cell (2007) 27(1):53–66. doi: 10.1016/j.molcel.2007.06.011 17612490

[75] Dufey E Bravo-San Pedro JM Eggers C Gonzalez-Quiroz M Urra H Sagredo AI. Genotoxic stress triggers the activation of IRE1alpha-dependent RNA decay to modulate the DNA damage response. Nat Commun (2020) 11(1):2401. doi: 10.1038/s41467-020-15694-y 32409639 PMC7224204

[76] Gonzalez-Quiroz M Blondel A Sagredo A Hetz C Chevet E Pedeux R. When endoplasmic reticulum proteostasis meets the DNA damage response. Trends Cell Biol (2020) 30(11):881–91. doi: 10.1016/j.tcb.2020.09.002 33036871

[77] Hetz C Zhang K Kaufman RJ. Mechanisms, regulation and functions of the unfolded protein response. Nat Rev Mol Cell Biol (2020) 21(8):421–38. doi: 10.1038/s41580-020-0250-z PMC8867924 32457508

[78] Yousefzadeh M Henpita C Vyas R Soto-Palma C Robbins P Niedernhofer L. DNA Damage-how and why we age? Elife (2021) 10. doi: 10.7554/eLife.62852 PMC7846274 33512317

[79] Costes S Langen R Gurlo T Matveyenko AV Butler PC. Beta-cell failure in type 2 diabetes: a case of asking too much of too few? Diabetes (2013) 62(2):327–35. doi: 10.2337/db12-1326 PMC3554362 23349537

[80] Wang J Wang H. Oxidative stress in pancreatic beta cell regeneration. Oxid Med Cell Longev (2017) 2017:1930261. doi: 10.1155/2017/1930261 28845211 PMC5560096

[81] Dhawan S Georgia S Bhushan A. Formation and regeneration of the endocrine pancreas. Curr Opin Cell Biol (2007) 19(6):634–45. doi: 10.1016/j.ceb.2007.09.015 PMC2695413 18061427

[82] Sturmlechner I Zhang C Sine CC van Deursen EJ Jeganathan KB Hamada N. p21 produces a bioactive secretome that places stressed cells under immunosurveillance. Science (2021) 374(6567):eabb3420. doi: 10.1126/science.abb3420 34709885 PMC8985214

[83] Banito A Lowe SW. A new development in senescence. Cell (2013) 155(5):977–8. doi: 10.1016/j.cell.2013.10.050 PMC4702512 24267881

[84] Demaria M Ohtani N Youssef SA Rodier F Toussaint W Mitchell JR. An essential role for senescent cells in optimal wound healing through secretion of PDGF-AA. Dev Cell (2014) 31(6):722–33. doi: 10.1016/j.devcel.2014.11.012 PMC4349629 25499914

[85] Munoz-Espin D Canamero M Maraver A Gomez-Lopez G Contreras J Murillo-Cuesta S. Programmed cell senescence during mammalian embryonic development. Cell (2013) 155(5):1104–18. doi: 10.1016/j.cell.2013.10.019 24238962

[86] Storer M Mas A Robert-Moreno A Pecoraro M Ortells MC Di Giacomo V. Senescence is a developmental mechanism that contributes to embryonic growth and patterning. Cell (2013) 155(5):1119–30. doi: 10.1016/j.cell.2013.10.041 24238961

[87] Van Gassen N Staels W Van Overmeire E De Groef S Sojoodi M Heremans Y. Concise review: macrophages: versatile gatekeepers during pancreatic beta-cell development, injury, and regeneration. Stem Cells Transl Med (2015) 4(6):555–63. doi: 10.5966/sctm.2014-0272 PMC4449100 25848123

[88] Homo-Delarche F. Immune cells: actors in pancreas development and regeneration that fail to fulfill their role and lead to diabetes? Discovery Med (2004) 4(23):344–50. 20704972

[89] Homo-Delarche F Drexhage HA. Immune cells, pancreas development, regeneration and type 1 diabetes. Trends Immunol (2004) 25(5):222–9. doi: 10.1016/j.it.2004.02.012 15099561

[90] Geutskens SB Otonkoski T Pulkkinen MA Drexhage HA Leenen PJ. Macrophages in the murine pancreas and their involvement in fetal endocrine development in vitro. J Leukoc Biol (2005) 78(4):845–52. doi: 10.1189/jlb.1004624 16037409

[91] Mussar K Pardike S Hohl TM Hardiman G Cirulli V Crisa L. A CCR2+ myeloid cell niche required for pancreatic beta cell growth. JCI Insight (2017) 2(15). doi: 10.1172/jci.insight.93834 PMC5543911 28768911

[92] Mussar K Tucker A McLennan L Gearhart A Jimenez-Caliani AJ Cirulli V. Macrophage/epithelium cross-talk regulates cell cycle progression and migration in pancreatic progenitors. PloS One (2014) 9(2):e89492. doi: 10.1371/journal.pone.0089492 24586821 PMC3929706

[93] Bonner-Weir S. Life and death of the pancreatic beta cells. Trends Endocrinol Metab (2000) 11(9):375–8. doi: 10.1016/S1043-2760(00)00305-2 11042468

[94] Georgia S Bhushan A. Beta cell replication is the primary mechanism for maintaining postnatal beta cell mass. J Clin Invest (2004) 114(7):963–8. doi: 10.1172/JCI22098 PMC518666 15467835

[95] Finegood DT Scaglia L Bonner-Weir S. Dynamics of beta-cell mass in the growing rat pancreas. estimation with a simple mathematical model. Diabetes (1995) 44(3):249–56. doi: 10.2337/diab.44.3.249 7883109

[96] Scaglia L Cahill CJ Finegood DT Bonner-Weir S. Apoptosis participates in the remodeling of the endocrine pancreas in the neonatal rat. Endocrinology (1997) 138(4):1736–41. doi: 10.1210/endo.138.4.5069 9075738

[97] Milanovic M Fan DNY Belenki D Dabritz JHM Zhao Z Yu Y. Senescence-associated reprogramming promotes cancer stemness. Nature (2018) 553(7686):96–100. doi: 10.1038/nature25167 29258294

[98] Li Y Zhao H Huang X Tang J Zhang S Li Y. Embryonic senescent cells re-enter cell cycle and contribute to tissues after birth. Cell Res (2018) 28(7):775–8. doi: 10.1038/s41422-018-0050-6 PMC6028486 29872106

[99] Trudeau JD Dutz JP Arany E Hill DJ Fieldus WE Finegood DT. Neonatal beta-cell apoptosis: a trigger for autoimmune diabetes? Diabetes (2000) 49(1):1–7. doi: 10.2337/diabetes.49.1.1 10615942

[100] Krischer JP Lynch KF Schatz DA Ilonen J Lernmark A Hagopian WA. The 6 year incidence of diabetes-associated autoantibodies in genetically at-risk children: the TEDDY study. Diabetologia (2015) 58(5):980–7. doi: 10.1007/s00125-015-3514-y PMC4393776 25660258

[101] Liu X Vehik K Huang Y Elding Larsson H Toppari J Ziegler AG. Distinct growth phases in early life associated with the risk of type 1 diabetes: the TEDDY study. Diabetes Care (2020) 43(3):556–62. doi: 10.2337/dc19-1670 PMC7035588 31896601

[102] Atkinson MA. The pathogenesis and natural history of type 1 diabetes. Cold Spring Harb Perspect Med (2012) 2(11). doi: 10.1101/cshperspect.a007641 PMC3543105 23125199

[103] Xu C Shen WB Reece EA Hasuwa H Harman C Kaushal S. Maternal diabetes induces senescence and neural tube defects sensitive to the senomorphic rapamycin. Sci Adv (2021) 7(27). doi: 10.1126/sciadv.abf5089 PMC8245044 34193422

[104] Thompson PJ Shah A Apostolopolou H Bhushan A. BET proteins are required for transcriptional activation of the senescent islet cell secretome in type 1 diabetes. Int J Mol Sci (2019) 20(19). doi: 10.3390/ijms20194776 PMC6801956 31561444

[105] Brawerman G Thompson PJ. Beta cell therapies for preventing type 1 diabetes: from bench to bedside. Biomolecules (2020) 10(12). doi: 10.3390/biom10121681 PMC7765619 33339173

[106] Wissler Gerdes EO Misra A Netto JME Tchkonia T Kirkland JL. Strategies for late phase preclinical and early clinical trials of senolytics. Mech Ageing Dev (2021) 200:111591. doi: 10.1016/j.mad.2021.111591 34699859 PMC8627448

[107] Gasek NS Kuchel GA Kirkland JL Xu M. Strategies for targeting senescent cells in human disease. Nat Aging (2021) 1(10):870–9. doi: 10.1038/s43587-021-00121-8 PMC8612694 34841261

[108] Criscione SW De Cecco M Siranosian B Zhang Y Kreiling JA Sedivy JM. Reorganization of chromosome architecture in replicative cellular senescence. Sci Adv (2016) 2(2):e1500882. doi: 10.1126/sciadv.1500882 26989773 PMC4788486

[109] Dhawan S Gurlo T Wang JK. 259-OR: epigenetic coregulation of beta-cell function and survival in postnatal life. Diabetes (2020) 69(Supplement 1):259–OR. doi: 10.2337/db20-259-OR

[110] Perea-Resa C Wattendorf L Marzouk S Blower MD. Cohesin: behind dynamic genome topology and gene expression reprogramming. Trends Cell Biol (2021) 31(9):760–73. doi: 10.1016/j.tcb.2021.03.005 PMC8364472 33766521

[111] Wang JK Pandey A Ap HAM Parveen N Dhawan S. 174-OR: ADA presidents’ select abstract: epigenetic regulation of functional beta-cell mass by cohesin Smc3. Diabetes (2021) 70(Supplement_1). doi: 10.2337/db21-174-OR

